# Co-Infections with Cytomegalovirus and Human Herpesvirus Type 7 in Adult Polish Allogeneic Haematopoietic Stem Cell Transplant Recipients

**DOI:** 10.1007/s00005-013-0252-z

**Published:** 2013-08-18

**Authors:** Agnieszka Tomaszewska, Anna Kryśko, Tomasz Dzieciątkowski, Maciej Przybylski, Grzegorz W. Basak, Kazimierz Hałaburda, Karolina Piekarska, Agata Sulowska, Barbara Nasiłowska-Adamska, Grażyna Młynarczyk, Wiesław W. Jędrzejczak, Bożena Mariańska

**Affiliations:** 1 Department of Haematopoetic Stem Cell Transplantation, Institute of Hematology and Transfusion Medicine, Warsaw, Poland; 2 Interfaculty Department of Biotechnology, Warsaw University of Life Sciences, Warsaw, Poland; 3 Department of Microbiology, Central Clinical Hospital in Warsaw, Warsaw, Poland; 4 Chair and Department of Medical Microbiology, Medical University of Warsaw, Chałubińskiego 5, 02-004 Warsaw, Poland; 5 Department of Hematology, Oncology and Internal Medicine, Medical University of Warsaw, Warsaw, Poland; 6 Second Faculty of Medicine, Medical University of Warsaw, Warsaw, Poland

**Keywords:** CMV, HHV-7, Haematopoietic stem cell transplantation, Infectious complications, Real-time PCR

## Abstract

Human herpesvirus 7 (HHV-7) is widespread around the world and may also be a possible cofactor for cytomegalovirus (CMV) infection in haematopoietic stem cell transplant (HSCT) recipients. In case of viral diseases where specific treatment is available, real-time PCR assays constitute reliable diagnostic tools enabling timely initiation of appropriate therapy and rapid assessment of the efficacy of antiviral treatment strategies. The presence of CMV and HHV-7 was confirmed by the detection of viral DNA isolated from 1,027 plasma samples. A group of 69 allogeneic HSCT (alloHSCT) recipients was examined in early post-transplant period using quantitative real-time PCR methods. Within the study period, 62 % of patients had at least once CMV DNA-emia, while HHV-7 DNA was found in 43 % of subjects. Co-infection between these β-herpesviruses was detected in the plasma samples collected from 18 patients (26 %). Patients with concomitant HHV-7 DNA-emia had significantly higher number of CMV DNA copies compared with those without HHV-7 infection (1986 vs. 432 copies/ml, *p* < 0.001) but there was no difference in duration of CMV DNA-emia between these groups. On the other hand, while the load of HHV-7 DNA was comparable between patients with CMV DNA-emia and without CMV DNA-emia, the duration of HHV-7 DNA-emia was significantly longer in the first group (38.5 vs. 14 days, *p* < 0.001). HHV-7 DNA-emia is very frequently detected in Polish alloHSCT recipients. In those, who have subsequent CMV reactivation, the coexistence of the viruses may negatively affect the kinetics of infection with either of them. Therefore the investigation of concomitant HHV-7 DNA-emia could affect the prognosis of post-transplant patients suffering from CMV reactivation.

## Introduction

The human β-herpesviruses–cytomegalovirus (CMV), human herpesviruses type 6 and 7 (HHV-6 and HHV-7)—are ubiquitous pathogens that infect the majority of humans. After primary infection, these viruses become latent in human host and are controlled by functioning immune system (Griffiths et al. 2000; Ljungman et al. 2008). Reactivation occurs usually in immunocompromised patients and can lead to illnesses which differ in clinical presentation from the disease associated with primary infection. Active CMV infection is known to be a major infectious complication after transplantation. On the other hand, the reactivation of HHV-7 virus is very rarely studied and its clinical significance remains still unknown (Ljungman et al. 2008). Some authors have postulated a potential increase in virulence of HHV-7 in the course of a simultaneous CMV reactivation, leading to a greater risk of CMV disease after transplantation (Chan et al. 1997; Chapenko et al. 2012; Zawilińska et al. 2011).

Only a few studies concerning comparison of CMV and HHV-7 DNA levels have been published so far. Therefore, the aims of the study were: (1) to determine the prevalence and viral load of HHV-7 DNA in adult allogeneic haematopoietic stem cell transplant (alloHSCT) recipients; and (2) to determine possible association between HHV-7 and CMV viraemia.

## Materials and Methods

This prospective study involved 69 patients who underwent an alloHSCT in the Department of Haematopoietic Stem Cell Transplantation, Institute of Hematology and Transfusion Medicine or in the Department of Hematology, Oncology and Internal Medicine, Medical University of Warsaw between June 2010 and July 2012. Monitoring of viral load in patients’ serum samples performed in the Department of Microbiology covered the early post-transplant period of 100 days after HSCT. The characteristics of patients are shown in Table 1.

**Table 1:** Characteristics of patients from the study group (*N* = 69)

Age (years)	43.6 ± 21
Gender (male/female)	36/33
Type of HSCT (related/unrelated)	27/42
Underlying haematological disease	
Acute myeloid leukemia	30
Acute lymphoblastic leukemia	11
Chronic myelogenous leukemia	8
Chronic lymphocytic leukemia	6
Multiple myeloma	3
Myelodysplastic syndrome	3
Lymphoma: Hodgkin/non-Hodgkin	3/3
Primary myelofibrosis	1
Waldenström’s macroglobulinemia	1

Collection of sera samples from all patients for PCR investigations began at a median of 3 days after transplantation (range 1–7 days) and lasted until a median of 92 days (range 28–105 days). Viral DNA presence was tested by quantitative real-time PCR (qPCR) in serum samples obtained once a week until the 100th day after allogeneic HSCT. The median number of samples per patient was 16 (range 3–28 samples) and a total of 1,027 samples were examined using real-time PCR methods. Viral DNA was extracted from the 200 μl of serum, using a High Pure Viral Nucleic Acid Kit^**®**^ (Roche Diagnostics, Germany) in accordance to the manufacturer’s instructions.

Cytomegalovirus DNA was detected using commercial CMV Quant Kit^**®**^ (Roche Diagnostics, Germany), according to the manufacturer’s protocol. For the detection of HHV-7, an in-house real-time PCR method was used, with HHV-7 DNA controls in the range 100–100,000 copies/ml (Dzieciatkowski et al. 2011). Both tests were run on the LightCycler 2.0 instrument (Roche Diagnostics, Germany) and each amplification reaction embraced, except tested samples and calibrators, a negative control of DNA extraction and amplification process.

Oral acyclovir in standard doses (400 mg/3 times a day) was used as an antiviral prophylaxis during the post-transplant period. If CMV DNA was detected using qPCR assay, the therapy with intravenous ganciclovir 5 mg/kg body weight every 12 h was administered (Ljungman et al. 2004).

Statistical analysis comprised the comparison of CMV and HHV-7 DNA load levels using Student’s *t* test. The Mann–Whitney rank sum test was used for comparing the onset and duration of the viraemia. The relationship between the risk of CMV and HHV-7 DNA-emia appearance was analyzed with *χ* ^2^ test. In all analyses, *p* value was set at the level of 0.001, due to a relatively small number of examined individuals. Statistical analysis was performed using SigmaStat 3.1 software package (Systat Software, Inc.).

## Results

The results of DNA of CMV and HHV-7 detection allowed to classify the patients into four logical groups: patients without CMV nor HHV-7 DNA-emia (*N* = 12); patients with concomitant CMV/HHV-7 DNA-emia (*N* = 18); patients who had only CMV DNA-emia (*N* = 26) and patients with HHV-7 DNA-emia alone (*N* = 13). As a result of amplification of DNA isolated from sera using CMV-specific qPCR assay, products were detected in samples taken from 44 individuals (64 %). In the majority of patients, CMV DNA-emia was found within the typical period of 30–70 days after transplantation and usually ranged between 800 and 10,000 copies/ml.

Positive results of HHV-7 real-time PCR were detected in samples taken from 30 patients (43 %). Three of them (4 %) had determined HHV-7 DNA presence in only single positive sample, while the remaining 27 patients (39 %) had positive result in two or more subsequent tests. The obtained quantitative results usually were at low to medium level, ranging between 200 and 3,000 copies/ml, and viraemia was observed between 20 and 60 days after transplantation.

Statistical analysis of the results revealed a significant difference (*p* < 0.001, Student’s *t* test) in CMV DNA-emia levels between two groups of patients, namely, patients with concomitant CMV/HHV-7 infection (average viral load in serum amounted to 1986 copies of CMV DNA per milliliter) and patients with CMV infection without detectable DNA of HHV-7 in sera samples (average CMV viral load 432 c/ml). No such difference was observed in HHV-7 DNA-emia levels (470 c/ml in group with CMV/HHV-7 DNA-emia vs. 204 c/ml in patients with HHV-7 alone; *p* = 0.01, Student’s *t* test).

A significant difference was also found in duration of HHV-7 viraemia in above mentioned groups of patients: in patients with simultaneous CMV/HHV-7 viraemia the median of HHV-7 DNA-emia duration amounted to 38.5 days, whereas in the group of patients without concomitant CMV viraemia, the median of HHV-7 DNA-emia duration was 14 days (*p* < 0.001, Mann–Whitney test). However, there was no statistical difference (*p* = 0.007, Mann–Whitney test) in the duration of CMV DNA-emia between the groups of patients with CMV alone (median: 21 days) and those with concurrent CMV/HHV-7 DNA-emia (median: 45 days).

In 94 % (17/18) of patients with concomitant CMV/HHV-7 viraemia, DNA of HHV-7 appeared before CMV (median: 10 days), but there was no significant difference in chance of CMV DNA-emia appearance, when compared with the group of patients which had CMV DNA-emia without corresponding presence of HHV-7 DNA in serum (*p* = 0.201, *χ* ^2^ test). Similarly, HHV-7 DNA-emia was not a predictive factor for subsequent development of CMV DNA-emia (*p* = 0.302, *χ* ^2^ test).

There were no difference between the median time of the onset of CMV and HHV-7 DNA-emia in the analyzed groups of patients (Table 2).

**Table 2:** Comparison of mean viral load, time of viraemia onset and viraemia duration in HSCT recipients with CMV/HHV-7 co-infection compared to patients infected with CMV or HHV-7 alone

	Concomitant infection of CMV and HHV-7 *N* = 18	CMV infection, w/o HHV-7 *N* = 26	HHV-7 infection, w/o CMV *N* = 13	*p* value
CMV average viral load in serum [log_10_ of DNA copies/ml]	3.298 (95 % CI: 0.188)	2.636 (95 % CI: 0.147)	N/A	<0.001^a,^*
Median of the onset of CMV viraemia [days after HSCT; lower/upper quartile]	42 (35–56)	42 (28–70)	N/A	0.625^b^
Median of the duration of CMV viraemia [days; lower/upper quartile]	45 (35–70)	21 (14–35)	N/A	0.007^b^
HHV-7 average viral load in serum [log_10_ of DNA copies/ml]	2.672 (95 % CI: 0.102)	N/A	2.31 (95 % CI: 0.165)	0.01^a^
Median of the onset of HHV-7 viraemia [days after HSCT; lower/upper quartile]	35 (28–35)	N/A	28 (24.5–42)	0.865^b^
Median of the duration of HHV-7 vireamia [days; lower/upper quartile]	38.5 (35–56)	N/A	14 (10.5–21)	<0.001^b,^*

*CI* confidence interval, *N/A* not applicable

* Statistically significant difference

^a^Analyzed with Student’s *t* test

^b^Analyzed with Mann–Whitney test

## Discussion

Monitoring and early treatment of various microbiological infections are critical in the management of patients after HSCT. Infections with widely spread herpesviruses are particularly likely to put the success of transplant at risk. Immediate detection and monitoring of β-herpesviruses plays an important role in the management of patients undergoing stem cell transplantation (Ljungman et al. 2008).

Available data suggest that HHV-7 infections are common after transplantation and may lead to CMV reactivation and cytomegalovirus disease (Chan et al. 1997; Chapenko et al. 2012; Kidd et al. 2000). In the present study, HHV-7 viraemia was found mostly (94 %) before CMV infection, which suggests a possible interaction between these β-herpesviruses. Our prior findings showed that HHV-7 viraemia may also occur simultaneously at the time of active infection caused by CMV (Dzieciatkowski et al. 2011). However, Humar et al. (2009) did not find evidence of potential interaction between CMV and HHV-7. Therefore, the question of interactions between the β-herpesviruses is still pending and requires further studies in larger group of patients.

Using real-time PCR we found that 43 % of our study group had HHV-7 in sera samples. This value is higher compared to some studies showing HHV-7 DNA-emia prevalence to be 13.6 % (Zawilińska et al. 2011) or 29.3 % (Humar et al. 2009), but similar to the value described by Hubacek et al. (2008) i.e. 44.8 %. HHV-7 was probably self-reactivated by the recipient as a result of such factors as a profound immune dysfunction or an allogeneic reaction after transplantation. This is only our hypothesis, due to the lack of commercial serological methods for measuring the presence of anti-HHV-7-antibodies.

We did not found clear association between HHV-7 viraemia and mortality during this study. The majority of HHV-7-positive patients had mild fever of unknown origin and two of them (7 %) had coexisting low respiratory tract disorders during the viraemia period. Importantly, infection with human HHV-7 may cause diagnostic problems, particularly mimicking some of the symptoms of skin form of graft-versus-host disease (GvHD). The relationship between HHV-7 and GvHD still remains unknown. Two of them may coexist, but the HHV-7 and/or CMV infection may also be a trigger for GvHD (Wang et al. 1996). From the other side, immunosuppression associated with GvHD and its therapy may increase probability of symptomatic herpesviral infection.

While acyclovir prophylaxis did not show any effect on HHV-7 DNA-emia, we found that the use of ganciclovir therapy for CMV disease was associated with a significant reduction of HHV-7 viral load. Nevertheless, two individuals developed HHV-7 viraemia during ganciclovir therapy.

In summary, using real-time PCR assays, we demonstrate the presence and the possible clinical relevance of co-infection of these two β-herpesviruses. There is a potential “priming” effect of HHV-7 reactivation leading to CMV positivity with adverse clinical effect on results of stem cell transplantation. Furthermore, the establishment of adequate procedures for HHV-7 detection and monitoring is advisable to clarify the significance of HHV-7 infection in HSCT recipients.

## Abbreviations

CMV: Cytomegalovirus
GvHD: Graft-versus-host disease
HHV-7: Human herpesvirus type 7
HSCT: Haematopoietic stem cell transplantation
qPCR: Quantitative real-time PCR
